# A case of generalized psoriasis-like skin lesions caused by rituximab use in a 14-years patient with systemic lupus erythematosus

**DOI:** 10.1186/1546-0096-9-S1-P249

**Published:** 2011-09-14

**Authors:** SR Rodionovskaya, IP Nikishina, IN Tsymbal, IN Lavrentieva

**Affiliations:** 1 Children's Hospital №38 Federal Medical Biological Agency of Russia, Moscow; 2 Research Institute of Rheumatology, Russian Academy of Medical Sciences, Moscow

## Background

Use of biologics in rheumatological patients can provoke psoriasis «de novo». It is well known phenomenon for TNF-ingibitors. We reported a serious incident of psoriasis-like skin lesions caused by rituximab use in a child with systemic lupus erythematosus (SLE).

## Case report

We have observed a 14-years-old female patient suffering from SLE during 4 years. She has, severe progressive nephritis with nephrotic syndrome (proteinuria 4 g/24 h), hematuria, hypoalbuminemia, high positive autoimmunity tests (ANA, anti DNA), IV class of lupus-nephritis by kidney biopsy. In the first year of disease the treatment of pulse therapy with methylprednisolone (MP) and cyclophosphamide was effective and resulted in remission of the disease. Relapse was developed after 4 years. MP Pulse therapy (6 procedures) was not successful. Rituximab therapy 375 mg/m^2^ (500 mg) was started. 2 days later the first infusion widespread papular rash without itching was appeared and persisting for 2 weeks. Then we observed febrile fever, widespread polymorphic rash (macular, papular, pustular, bullous), ESR 90 mm/hour, CRP 60 mg/l, proteinuria 3.7-4.1 g/24. Procalcitonin test was negative. Plating of pustules secretions was sterile. Skin biopsy revealed the phenomenon of hyperkeratosis, exfoliation of superficial layers of epidermis. Antibiotic and antihistamine therapy were ineffective. It was increased the dose of methylprednisolone to 1.3 mg/kg, cyclosporine A and topical skin steroids were added. Rituximab was canceled. The treatment was result in positive effects: reduction of proteinuria, hematuria and cutaneous manifestations.

**Figure 1:**
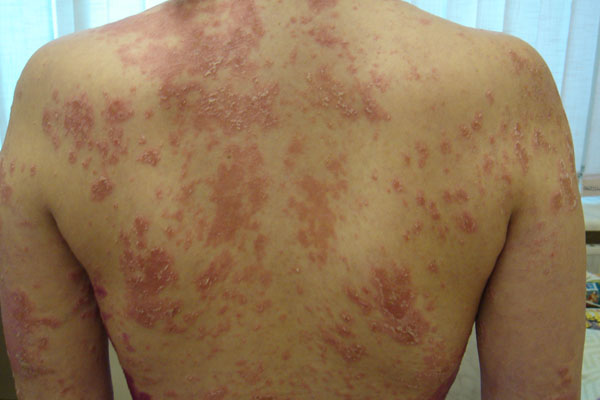


## Conclusions

Rituximab therapy in autoimmune diseases may induce unusual psoriasis-like reactions.

